# Effects of Resistance Exercise on Balance Ability: Systematic Review and Meta-Analysis of Randomized Controlled Trials

**DOI:** 10.3390/life10110284

**Published:** 2020-11-15

**Authors:** Nejc Šarabon, Žiga Kozinc

**Affiliations:** 1 Faculty of Health Sciences, University of Primorska, 6310 Izola, Slovenia; ziga.kozinc@fvz.upr.si; 2 Laboratory for Motor Control and Motor Behavior, S2P, Science to Practice, Ltd., 1000 Ljubljana, Slovenia; 3 InnoRenew CoE, 6310 Izola, Slovenia; 4 Andrej Marušič Institute, University of Primorska, 6000 Koper, Slovenia

**Keywords:** balance, postural control, strength training, resistance exercise, health

## Abstract

With this systematic review, we explored whether resistance exercise (RE) could be used to improve balance in addition to muscular strength and power. Scientific databases were searched for randomized controlled trials that investigated the effects of RE on the performance of various balance tests. Studies were considered if they involved healthy participants of any age group. Thirteen studies were included in the meta-analysis. The results showed moderate to large improvements in balance ability following RE in older adults, as reflected in functional reach test (mean difference (MD): +4.22 cm, *p* < 0.001), single-leg standing test (MD: +1.9–37.6 s, *p* < 0.001) and timed-up-and-go test (MD: −0.55 s; *p* = 0.002). Moderate to large improvements following RE were seen in adults in star excursion balance test (MD: +4.09–5.17 cm; *p* = 0.001–0.020), but not for Y-balance test score (MD: +4.94%, *p* = 0.14). The results implicate that RE interventions may significantly improve balance ability in adults and older adults. Therefore, RE could be used to improve balance in these populations, while further studies are needed to investigate children populations. Performing RE alone could be a time-efficient compromise for individuals who are unwilling or unable to perform large volumes of exercise or different exercise modalities.

## 1. Introduction

Regular physical activity is known to have numerous immediate and long-term benefits for an individual and the society, spanning from (but not limited to) reduced mortality [1], higher quality of life [2,3,4], increased independence, and reduced risk of falls in older adults [5,6], improved cognitive abilities [7,8], and reduced incidence of several chronic non-communicable diseases [9,10]. A considerable amount of work has been committed to providing the best physical activity guidelines to obtain these benefits [11,12]. Typically, a combination of different exercise modalities (e.g., resistance exercise, aerobic activities, flexibility training, balance exercises) are advised [11,12]. With that in mind, the World Health Organization has been stressing that one in four adults are not active enough to elicit significant health benefits, and up to 80% of the World’s adolescent population is insufficiently physically active [13]. Indeed, most recent investigations are showing a very similar picture [14,15].

The literature is abundant with studies that explored the barriers and facilitators of physical activity in different populations [16,17,18], which is helpful to practitioners and researchers for designing interventions and training programs. Due to the unwillingness or poor motivation of many individuals to perform regular high-volume exercise, it is also reasonable to explore which types of exercise have the largest overall effect on health-related outcomes. For instance, resistance exercise (RE) and balance/stability training are both advocated as important components for improving physical function in the elderly [5,6], and both muscle strength and postural control are paramount for athletic performance [19,20] and efficiency and independence in everyday life [21]. If the training of one of these abilities produces a positive effect on the other, it could be put forward as a primary recommendation for individuals who are willing to perform the only limited volume of exercise. In this review, we investigated whether RE training elicits improvements in balance ability. As the sufficient balance/postural control ability is desired for all populations, especially for the elderly in connection to reduce the likelihood of falls [22] and increased independence in activities of daily life [23], it is practically and clinically useful to explore alternative interventions that could improve balance, other than interventions targeting balance and stability directly. Such interventions could promote better overall function and well-being, which is especially crucial for individuals who are not willing to perform a lot of exercise. RE is promoted as one of the most important measures to prevent sarcopenia [24]. If RE is also beneficial for balance in adults, it could also be recommended for them as the primary exercise modality choice when their time is restricted, and it could help prevent (later in life) both sarcopenia [24] and problems, associated with poor balance. 

It has been shown that individual physical abilities are positively related. For instance, Wilson et al. [25] have reported that isometric hip strength is positively related to Y-balance test scores in healthy participants. Furthermore, quadriceps strength has been shown to be associated with the limits of stability in young women [26]. However, strength and balance may not be related in older adults [27]. While this observation suggests that multi-component programs could be the optimal approach to improve the health of older adults, it could be more time and cost-efficient to introduce exercise modalities that promote the broadest effects; i.e., one type of training promoting the increase of more than one physical ability. Indeed, it has been suggested that RE may promote an increase in balance ability in addition to increases in strength and power, both in the adult [28,29,30] and older populations [31,32,33,34]. Specifically, moderate to large effects of RE were found for various dynamic balance and mobility tests, such as functional reach test [32,33], timed-up-and-go test [34], and single-leg standing test [33]. These studies have already indicated some important characteristics of RE that need to be considered when the purpose is to improve the balance. For instance, Shiotsu et al. [33] reported improvements in various functional balance tests in older adults after high-intensity (60–70% 1RM) RE, but not after lower intensity (40–50% 1RM) RE. Moreover, Asadi et al. [30] have demonstrated very large effects of plyometric RE on star-excursion balance test scores in adults. 

While the current evidence suggests that balance could be improved with RE interventions, to the best of our knowledge, the studies that examined the effects of RE on balance ability have not been systematically reviewed before. Therefore, the purpose of this paper was to provide a systematic review and meta-analysis of randomized controlled trials (RCT) that investigated the effects of different RE interventions on balance ability across all age groups. We hypothesized that RE will have positive effects on balance ability in children, adults, and elderly populations. The secondary aim was to analyze the effects of various moderator variables on the observed effects of RE. These variables included participant characteristics (age, gender, body composition) and intervention characteristics (duration, intensity, volume, type, etc.) 

## 2. Materials and Methods

The review process was registered a priori to the PROSPERO database (ID: CRD42020178520) and followed the PRISMA guidelines [35]. 

### 2.1. Inclusion and Exclusion Criteria

Study inclusion and exclusion criteria were structured according to the PICOS tool [36]:

- **Population (P):** The population of interest included all age groups and both genders. Therefore, children and adolescents (≤18 years of age), adults (18–65 years of age), and older adults (≥65 years of age) were considered. Studies were excluded if they included participants with neurological (e.g., Parkinson’s disease, multiple sclerosis) or musculoskeletal conditions (e.g., osteoarthritis, choric pain).
- **Intervention (I):** Studies were included if at least one experimental group performed an intervention consisting of RE. We imposed no limits regarding the RE type (e.g., maximal strength training, speed-power training, strength endurance training), regarding the type of the load used (e.g., elastic, free weight, machine, bodyweight). No exclusion criteria were determined for different RE intensity or volume, however, we excluded the interventions lasting <3 weeks. Studies were also excluded in case the RE was conducted in conjunction with other exercise modalities. In cases when another non-exercise intervention was used (e.g., nutritional supplement), the study was included if it involved a control group that did not perform the RE but received the respective non-exercise intervention.
- **Comparisons (C):** The main inclusion criteria were an inclusion of a control group, that received no RE intervention. The exceptions (see also above) were possible when both experimental and control groups received an additional non-exercise intervention (e.g., if the experimental group included RE and nutritional supplement, a control group was only considered if only the supplement was used).
- **Outcomes (O):** The studies were considered if the outcome measures included any type of functional balance/postural control test, or dynamic mobility test scores (including, but not limited to functional reach test, timed-up-and-go test, single-leg stance test, Y-balance test, star-excursion balance test, Romberg test). The mobility tests, such as the timed-up-and-go test were included because it was expected that such tests are commonly performed in the elderly populations, and although these tests are not stressing the balance ability, they were shown to be associated with the risk of falling in elderly and to reflect the balance ability in addition to mobility [37].
- **Study design (S):** To provide the highest quality of evidence, RCTs that included at least one RE intervention group and control group were considered.

### 2.2. Search Strategy

Multiple databases of scientific literature (PubMed, Cochrane Central Register of Controlled Trials, PEDro and ScienceDirect) were searched in April 2020 with no date restrictions. For the databases that enable using Boolean search operators, we used two combinations of search keywords, with the first being: *(*“*resistance exercise*” *OR* “*strength training*” *OR* “*power training*” *OR* “*weight lifting*” *OR* “*muscle strengthening*”*) AND (*“*star excursion*” *OR* “*Y-test*” *OR* “*Y test*” *OR* “*Y-balance test*” *OR* “*Romberg test*” *OR* “*single-leg stance*” *OR* “*functional reach test*” *OR* “*limits of stability*” *OR* “*limits-of-stability*” *OR* “*timed up and go*”*).* After a preliminary search, it was found that including “balance” as a keyword resulted in an unmanageable number of papers due to the increased number of papers not related to exercise at all. Therefore, we opted for a more specific combination of keywords, related to specific balance tests. Because such an approach could potentially miss out on relevant studies, we additionally scrutinized the reference lists of several review articles describing resistance exercise interventions. Finally, we carefully reviewed reference lists of all articles that were already retrieved through the database search. The database search was performed independently by two authors (NS and ZK). Two authors (NS and ZK) also screened the titles and the abstracts independently. Potentially relevant articles were screened in full text. 

### 2.3. Data Extraction

The data extraction was performed independently by two authors (NS and ZK) and disagreements were resolved through additional revision and consultation. The extracted data included: (a) baseline and post-intervention means and standard deviations for all eligible outcome measures (i.e., balance test scores) for interventional and control groups (b) baseline demographics of participants (gender, age, body height, body mass) (c) intervention characteristics (target body area (upper, lower, or whole-body), duration of the intervention, number of sessions per week, volume (number of exercises, sets, and repetitions), breaks between exercises and sets, supervision, and progression of exercise difficulty). Data was carefully entered into Microsoft Excel 2016 (Microsoft, Redmond, WA, USA). In case the data was presented in a graphical rather than tabular form, we used Adobe Illustrator Software (version CS4, Adobe Inc., San Jose, CA, USA) to determine the means and standard deviations. In case of missing data, the corresponding author of the respective article was contacted by e-mail. If there was no response after 21 days, the author was contacted again. If the author did not reply to the second inquiry, the data was considered to be irretrievable. 

### 2.4. Data Analysis and Synthesis

The data analysis was carried out in Review Manager software (Version 5.3, The Nordic Cochrane Centre, The Cochrane Collaboration, Copenhagen, Denmark, 2014). Before the results were entered into the meta-analytical model, the pre-post differences were calculated by subtracting the baseline value from the post-intervention value. The pooled standard deviations were calculated according to the following formula SD = √ [(SD_pre_^2^ + SD_post_^2^) − (2 × r × SD_pre_ × SD_post_). The correction value (r), which represents the pretest–posttest correlation of outcome measures, was conservatively set at 0.75. It should be noted that a change in the correction value in the range between 0.5 and 0.9 had little effect on the final pooled SD and would not change the outcomes. For the meta-analyses, the inverse variance method for continuous outcomes with the random-effects model was used. The pooled effect sizes were expressed as mean difference (MD) where possible, which allows the effect size to be expressed in units of measurement, specific for each test. Where this was not possible due to the heterogeneity of the studies the effect sizes were expressed as standardized mean difference (SMD). For MD and SMD, the respective 95% confidence intervals were also calculated and reported. SMD was interpreted as very small (<0.2), small (0.2–0.5), moderate (0.5–0.8), and large (>0.8) [38]. Statistical heterogeneity among studies was determined by calculating the I^2^ statistics. According to Cochrane guidelines, the I^2^ statistics of 0% to 40% might not be important, 30% to 60% may represent moderate heterogeneity, 50% to 90% may represent substantial heterogeneity and 75% to 100% indicates considerable heterogeneity [39]. The threshold for statistical significance was set at *p* ≤ 0.05 for all analyses. 

### 2.5. Assessment of Study Quality

The authors evaluated the quality of the studies using the PEDro scale, which assesses study quality based on ten criteria [40]. Potential disagreements between ratings were resolved by consulting. Studies scoring from 9–10 scores were considered as “excellent”, 6–8 as “good”, 4–5 as “fair” and less than 4 as “poor” quality. The PEDro scale was chosen because it was developed specifically to assess the quality of randomized controlled trial studies evaluating physical therapist interventions [40]. 

## 3. Results

### 3.1. Search Results

**Figure 1:**
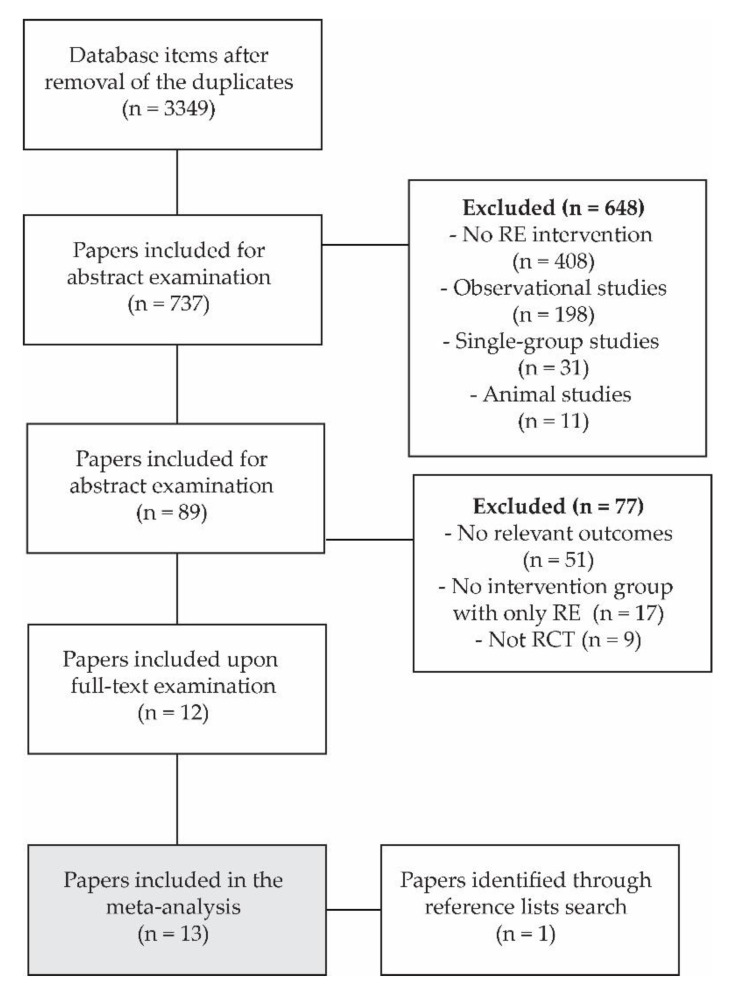
Flowchart of the study search and selection.

The study search and selection are summarized in a flowchart in Figure 1. Across all databases, 3349 articles were identified. Based on the screening of the title, 737 papers were included for abstract examination, after which 89 papers remained as potential candidates for meta-analyses. After full-text examinations, 12 studies (13 experimental groups) were included for the final meta-analysis, and 1 more study was retrieved through the reference list search. Therefore, 13 studies (14 experimental groups) were included in the meta-analysis. The population, intervention descriptions, and main outcomes of each study are summarized in Table 1 and Table 2. In 7 studies (8 experimental groups), the participants were older adults, while 5 studies examined adults and 1 study examined children. In addition, 2 studies met all of the inclusion criteria (1 examining older adults [31] and 1 examining adults [29]), but the outcome measures could not be pooled with the results of any of the other studies. The results of these two studies are summarized in Table 1 and Table 2 but are not included in the meta-analyses.

In the studies that examined older adults, 6 experimental groups consisted of participants of both genders, and 2 experimental groups consisted of females only. In total, there were 137 participants in the experimental groups (pooled demographic data: 71.2 ± 4.8 years of age; 66.8 ± 11.8 kg body mass, 160.8 ± 6.9 cm body height) and 117 participants in the control groups (pooled demographic data: 72.3 ± 5.1 years of age; 70.6 ± 10.8 kg body mass, 160.9 ± 7.8 cm body height). The RE was supervised in 5 of 7 studies. In the studies that examined children and adults, 5 experimental groups consisted of participants of both genders, and 1 experimental group consisted of males only. In total, there were 74 participants in the experimental groups (pooled demographic data: 19.3 ± 1.4 years of age; 65.7 ± 10.9 kg body mass, 166.8 ± 8.9 cm body height) and 72 participants in the control groups (pooled demographic data: 19.3 ± 1.3 years of age; 64.2 ± 10.4 kg body mass, 160.9 ± 7.8 cm body height). The RE was supervised in all the 6 studies.

The PEDro scale scores showed fair to good quality of the studies conducted on older adults (mean = 6.0 ± 0.58; median = 6.0; range = 5–7) and studies conducted on adults (mean = 5.33 ± 0.82; median = 5.5; range = 4–6). The items that almost all studies failed to satisfy were blinding of the subjects, therapists, and assessors.

### 3.2. Meta-Analyses

**Figure 2:**
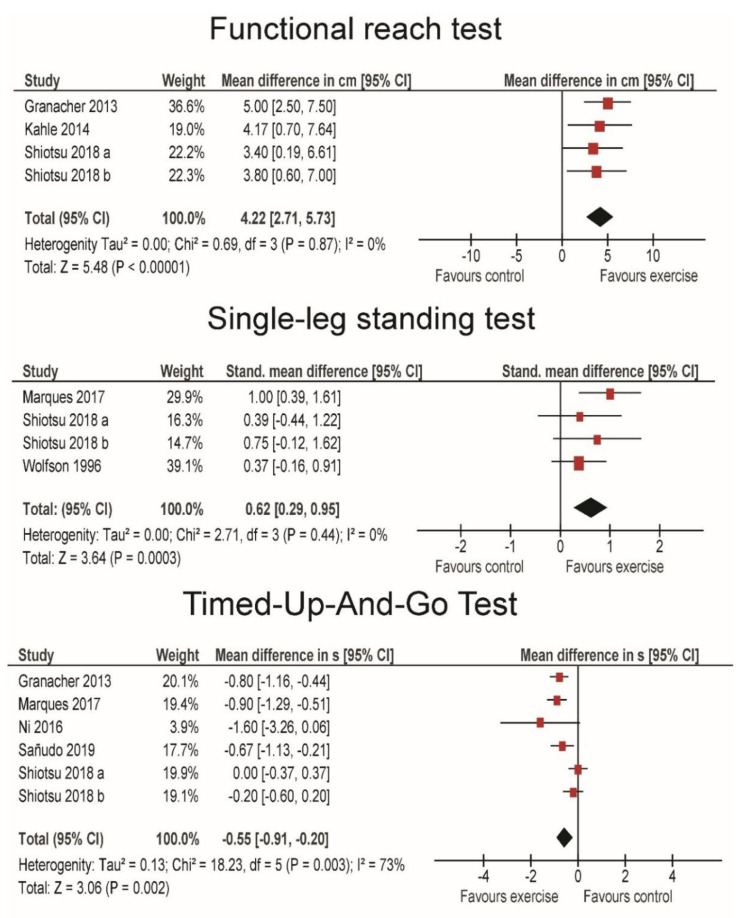
Forrest plots showing the pooled results for older adult populations.

Figure 2 shows the pooled results from the studies that examined older adult participants. The studies showed statistically significant effect positive of RE on functional reach test (MD = 4.22 cm; *p* < 0.001). The findings were very consistent across studies (I^2^ = 0%) and the magnitude of the effect was large (SMD = 1.04; 95% CI = 0.62–1.46). Due to the high heterogeneity between the studies (I^2^ = 72%) in terms of a single-leg standing test, only SMD was computed for this test and showed statistically significant moderate improvements with resistance exercise (SMD = 0.62; 95% CI = 0.29–0.95; *p* < 0.001). The absolute pre-post difference ranged from 1.9 s to 37.6 s across studies. Finally, there was also a statistically significant improvement in timed-up-and-go test (MD = −0.55 s; 95% CI = −0.91–−0.20; *p* = 0.002). The effects were large (SMD = 0.84; 95% CI = 0.40–1.28), although the studies were somewhat heterogeneous (I^2^ = 50%).

**Figure 3:**
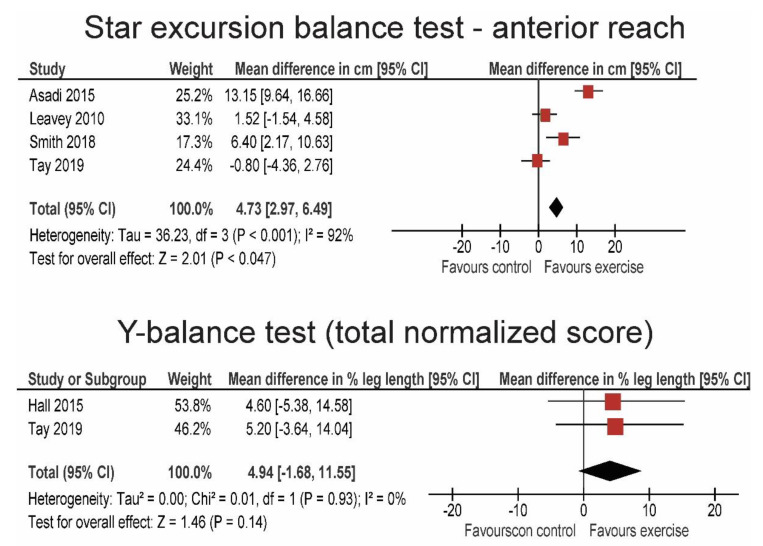
Forrest plots showing the pooled results for the adult population.

Figure 3 shows the pooled results from the studies that examined adults. Statistically significant improvements in the star excursion balance test were seen across studies. Specifically, the anterior reach was increased for 4.73 cm (95% CI = 2.97–5.49; *p* = 0.047). The effect size was large (SMD = 1.20; 95% CI = 0.34–2.08), however, the studies were not very consistent (I^2^ = 92%). Slightly smaller (MD = 4.01 and 4.16 cm; SMD = 0.72 and 0.73), but still statistically significant (*p* = 0.004–0.031) effects were observed for posterior-medial and posterior-lateral directions, respectively. Moreover, the pooled results from two studies showed a positive trend for total (i.e., the sum of the three directions) Y-balance test score. Specifically, the leg-length-normalized score increased for 4.94% (95% CI = −1.68–11.55%), which was not statistically significant (*p* = 0.14). The two studies were very consistent, showing almost identical results (I^2^ = 0%). 

## 4. Discussion

The purpose of this paper was to systematically review the RCT studies that explored the effects of RE on balance ability. We found that studies have consistently shown moderate to large improvements in balance ability following RE in adults and older adults, whereas only one study in children was found. It should also be noted that within the adult subgroup, all studies involved young adults (age 19–23). Nevertheless, these results imply that RE could offer an alternative approach to improve balance in adult and older participants. The secondary aim of this study was to explore the effects of various moderator variables, such as participant characteristics (age, gender, body composition) and intervention characteristics (duration, intensity, volume, type, etc.). Due to the small number of studies with common outcome measures, the appropriate statistical procedures (subgroup analyses or meta-regressions) could not be performed. Therefore, only a limited qualitative discussion is provided later in the discussion regarding the effects of moderator variables. 

Previous studies have shown that strength and balance are related [25,26] and that RE may elicit significant improvements in balance ability [27,28,29,31]. This systematic review supports this by providing strong evidence from RCT studies. Therefore, RE could be an optimal choice when various barriers [16,17,18] prevent the individuals to engage in large volumes of exercise. This might be particularly important for older adults because physical exercise is paramount for them to prevent falls and maintain independence [5,6]. The performance of tests included in the meta-analysis (i.e., the functional reach test, single-leg stance, and timed-up-and-go test) are all indicative of the risk of falls [22,49]. The improvements in the balance test were consistent across studies, even though the baseline ability of the participants varied across studies. The consistent moderate or large improvements of scores seen in the present systematic review implies the RE substantially lowers the risk of falls. Indeed, RE has been directly shown to decrease the risk of falls, either as a standalone intervention or as a part of multi-component interventions [5]. Shiotsu et al. [33] reported that the balance ability of older adults (as assessed through functional reach test, single-leg standing, and timed up and go test) was improved after moderate-intensity, but not low-intensity RE intervention. Therefore, moderate- and possibly high-intensity RE is likely superior to low-intensity RE in terms of the effect on balance. Moreover, an abundance of recent studies has shown that speed-power training elicits greater positive effects on the physical ability and function of older adults, compared to traditional strength training [50]. 

There is more than one possible underlying mechanism for the improvements in balance seen after RE. One of the simplest explanations is that most balance tests require at least some level of strength, which could be particularly evident for older adults. The second explanation could be that RE introduces some level of instability that must be compensated by the body. Previous studies have shown stability-specific strength gains [51] when comparing RE with different levels of instability (e.g., machines as a low instability approach; free weights as high instability approach). However, there were not enough studies in the present review to compare the effects of different RE types on balance ability. Overall, the improvements seemed to be elicited by RE regardless of the instability. Similar tasks-specificity is also present for stability and balance exercises (i.e., the most pronounced exercise effects are observed within the tasks used during training) [52]. Perhaps even more surprisingly, balance training alone has also been reported to elicit significant strength gains in adult [53] and older adult participants [54], and even in the rate of force development [55]. It seems however, that such effect are only seen when participants are untrained, as it was noted that these effects are absent when the balance training is preceded by a period of strength training [55] and that there are is no additional effect of balance training performed concomitantly with RE [56]. The exact underlying mechanism behind the concomitant improvements in strength and balance remains elusive, though previous studies have implicated the involvement of changes in corticospinal excitability [52]. Moreover, it was shown that RE reduces intracortical inhibitory networks within the primary motor cortex (M1) and corticospinal pathway [57] and increases the propagation velocity of action potentials along the muscle fibers [58], which could in turn influence automatic and voluntary muscle actions for maintaining balance. 

It should be noted that the interaction between the RE and improvements does not imply that RE should be always used as a preferential mode of exercise. For instance, RE is advised in combination with aerobic exercise in patients with cardiovascular diseases or risk factors [59], and RE must be used with caution in some cases, such as with hypertension patients [59,60]. Moreover, multi-component exercises programs are likely more effective than single-component programs [5,61]. The results of this study only imply that performing RE alone offers a good approach which will likely balance ability. With that in mind, future studies should address the remaining questions, such as the effects of exercise intensity, the type of the load, and the type of muscular contraction on balance ability and possibly other motor abilities in different populations. The specific effects of high-intensity exercise [50] and eccentric exercise [62] on muscular strength and physical function have already been addressed, however, it remains unknown how different forms of RE influence balance and other motor abilities. In light of task-specific adaptations to training [52], it could be speculated that RE under unstable conditions could bring the best of both worlds. These alternative forms of RE were omitted in this review, however, there is some evidence that training on unstable surfaces could induce significant and concomitant improvements in muscle strength, power, and balance in healthy older adults [63]. In summary, the choice of exercise modality should be carefully planned based on the available individual’s time, his/her physical status and motivation, while acknowledging both task-specificity of adaptations, as well as expected transfers of training effects to different motor abilities. 

The secondary aim of this study was to explore the effects of various moderator variables, such as participant or intervention characteristics. Due to a smaller number of studies, only a limited qualitative discussion is provided here. Most studies included participants of both genders, which suggests that both females and males improved balance ability with RE. Within older adult studies, 2 studies (3 experimental groups) consisted of female participants. There was no clear trend for the effect of gender regarding functional reach test, however, in one of the studies (2 experimental groups) with female participants, there were no improvements in timed-up-and-go tests. One study on older adults included only males and reported the highest increases in star-excursion balance tests among all included studies. However, it should be noted that this study was also very specific in other aspects, such as being the only one to include plyometric exercise [30]. Collectively, our results suggest that both genders improved balance with RE, with a possibility of smaller effects in females. 

The participants’ age was very consistent in both subgroups; therefore, it is very difficult to even speculate whether the age is an important factor for the observed effects. One study stood out slightly with somewhat older participants (80.0 ± 4.1 years) and showed smaller effects of RT on a single-leg standing test score compared to some other studies conducted with older adults. However, the same study also had a shorter training period (only 3 weeks), which is a more likely explanation for lower effects. In the adult subgroup, most interventions lasted for 6 weeks, except one lasting 4 weeks, therefore, no indication of intervention duration could be deduced. 

Because the magnitude of the effects was mostly quite consistent across studies, it is impossible to determine the effects of type of load, exercise intensity and volume, weekly frequency, and targeted muscle groups. As noted, one study of shorter duration (3 weeks) showed lower effects, which indicates that interventions of longer durations should be conducted. A study by Shiotsu et al. [33] implicated that intensity should not be too low, as their group that used lower intensity (40–50% 1RM) improved less in a single-leg standing test than the group that used higher loads (60–70% 1RM). No other indication for the effect of intensity was observed. It could be that, at least in adults, plyometric exercise is more effective compared to strength, power, or strength-endurance exercise modalities. Namely, the study by Asadi et al. [30] reported by far the largest improvements in star excursion balance tests. While we could not statistically compare different interventions, it seems that different RE modalities (free weights, bodyweight exercises, elastic exercise, and resistance machines) are all effective. Moreover, we found evidence for improvements in the balance following RE that was targeted at the full-body, only lower limbs, only trunk, and lower limbs in combination with the trunk, and even for RE targeting a single joint (ankle). Therefore, combining lower limb and trunk RE is likely the best for improvements in balance. Most of the interventions were performed 2–3 times per week, which is in accordance with the current guidelines for RE [11,12]. Collectively, most of the examined studies presented a positive effect of RE on balance ability, regardless of the intervention duration, load type, or other characteristics. At this point, it is impossible to reliably determine the optimal RE training for improving balance. 

### Limitations

Several limitations of the present systematic review need to be acknowledged. Firstly, only RCT studies were included. Although this was done to ensure that only the studies of the highest quality will be included, some important evidence from the remaining studies could be overlooked. Moreover, a small number of studies and different assessment tests prevented us to preform subgroup analyses, which would be valuable to assess the effects of different independent variables, such as exercise intensity, weekly frequency, and baseline physical activity, and physical status of the participants. No studies were found that were conducted with adult participants in the age range between 30 in 65 years. While the effects of RE on balance were very consistent in younger adults (18–25 years of age) and older participants (≥65 years of age), the findings cannot be generalized to the adult whole population. Moreover, only one study conducted on children was included, and although it found statistically significant improvements in star-excursion balance tests [28], the evidence regarding the effects of RE on balance ability is lacking. Finally, this review does not reveal whether the RE-induced effect on balance is effective for the reduction of incidence of falls. Although both RE and balance exercises are purported to reduce the risk of falling [64,65], and although poor postural balance is linked to falls [22,49], we cannot know whether improvements in balance, achieved through RE, are effective for reduction of falls. 

## 5. Conclusions

This systematic review has shown that RE interventions may significantly improve balance ability in adult and older adult participants. This finding has important practical implications, as RE could be used to improve both muscular strength and power, as well as balance at the same time. Strength training, power training, and strength-endurance training targeting primarily lower limb musculature, with different types of load (bodyweight, elastic, free weight, and resistance machines) were shown to be effective. RE interventions designed for improvements in balance ability should be sufficiently long (4 weeks and more) and be conducted in accordance with general RE. Although multi-component exercise programs should still be prioritized when possible, performing RE alone could be a time-efficient compromise when trying to improve overall physical fitness. 

## Figures and Tables

**Table 1:** Characteristics of the included studies with older adult participants.

Study	Sample Gender and Age	Intervention	Results
Granacher, 2013 [41]	Mixed (70.8 ± 4.1)	Strength endurance exercise, 9 weeks, 2 × week, 60 min, 3–4 sets, 15–20 repetitions, bodyweight exercises for trunk muscles.	↑ TUG (−0.4 s); ↑ FRT (+6.5 cm)
Hamed, 2018 [31]	Mixed (70.6 ± 3.1)	Strength endurance exercise, 14 weeks, 2 × week, 90 min, 3 sets, 10–20 repetitions, 20–85% 1RM, resistance machine exercises for trunk, hip, knee, and ankle muscles.	No change in limits of stability score (−0.3 cm)
Kahle, 2014 [32]	Mixed (75.6 ± 3.6)	Strength training, 6 weeks, 3 × week, 20 min, 1 set, 10–12 repetitions, bodyweight exercises for trunk and hip muscles.	↑ FRT (+4.6 cm)
Marques, 2017 [42]	Female (67.0 ± 5.0)	Strength training, 32 weeks, 3 × week, 50–80% 1RM, full-body training with resistance machines.	↑ TUG (−0.66 s); ↑ SLS test (+6.57 s);
Ni, 2016 [43]	Mixed (71.6 ± 6.6)	Strength and power training, 12 weeks, 2 × weekly, 45–60 min, 50–70% 1RM, mixed load compound exercises for lower and upper limbs.	↑ TUG (−1.3 s);
Sanudo, 2019 [34]	Mixed (64.4 ± 3.61)	Strength training, 6 weeks, 2–3 × week, 4 sets, 9 repetitions, flywheel resistance squat exercise.	↑ TUG (−0.83 s);
Shiotsu, 2018a [33]	Female (70.4 ± 4.1)	Low-intensity strength training, 10 weeks, 2 × week, 20 min, 3 sets, 10–15 repetitions, 40–50% 1RM, full-body workout with resistance machines.	↑ TUG (−0.2 s); ↑ FRT (+3.8 cm) ↑ SLS test (+9.5 s);
Shiotsu, 2018b [33]	Females (69.6 ± 4.6)	Strength training, 10 weeks, 2 × week, 20 min, 3 sets, 6–8 repetitions, 60–70% 1RM, full-body workout with resistance machines.	↑ TUG (−0.4 s); ↑ FRT (+4.2 cm) ↑ SLS test (+28.7 s);
Wolfson, 1996 [44]	Mixed (80.0 ± 4.1)	Strength training, 3 weeks, 3 × week, 45 min, 2 sets, 10–15 repetitions, 70–75% 1RM. Bodyweight, resistance machine, and free weight exercises for hip, knee, and ankle muscles.	↑ SLS test (+0.9 s);

TUG—timed-up-and-go test; FRT—functional reach test, SLS—single-leg standing test; 1RM—one-repetition maximum. ↑—increase of the respective outcome measure

**Table 2:** Characteristics of the included studies with adult and children participants.

First Author, Year	Mean Sample Age	Intervention	Results
Hall, 2015 [45]	Mixed (19.7 ± 2.2)	Strength endurance exercise, 6 weeks, 3 × week, 2–4 sets, 10–15 repetitions. Elastic resistance exercise for ankle muscles.	↑ total Y-test score (+4.6 cm)
Tay, 2019 [46]	Mixed (23.0 ± 1.7)	Power training, 6 weeks, 2 × week, 30 min, 3 sets, 8 repetitions, intensity 7/10 RPE, resistance machine, and free-weight exercises for lower limbs and trunk	↑ total Y-test score (+5.2 cm) ↑ SEBT scores (A: +4.1 cm. PM: +5.5 cm. PL: +6 cm)
Asadi, 2015 [30]	Male (20.1 ± 0.8)	Speed-power training, 6 weeks, 2 × week, 60 min, 3 sets, 20 repetitions, bodyweight plyometric exercises (various jumps).	↑ SEBT scores (A: +13.83 cm. PM: +3.12 cm. PL: +2.53 cm)
Leavey, 2010 [47]	Mixed (22.1 ± 1.5)	Power training, 6 weeks, 3 × week, 3–4 sets, 10–20 repetitions, 50–70% 1RM. Bodyweight and elastic resistance exercises for lower limbs, with most focus on hip abductors.	↑ SEBT scores (A: +1.53 cm. PM: +3.38 cm. PL: +6.24 cm)
Ozmen, 2016 [28]	Mixed (10.9 ± 0.3)	Power training, 6 weeks, 2 × week, 1 set, 20 repetitions, bodyweight exercises for trunk and lower limbs, focusing on abdominal, low-back, and pelvic muscles.	↑ SEBT scores (A: +7.47 cm. PM: +5.69 cm. PL: +4.78 cm)
Smith, 2018 [48]	Mixed (20.1 ± 1.7)	Strength endurance exercise, 4 weeks, 3 × week, 3 sets, 20 repetitions, elastic resistance exercises for hip muscles.	↑ SEBT scores (A: +7.4 cm. PM: +9.4 cm. PL: +12.4 cm)
Yoo, 2018 [29]	Mixed (19.2 ± 0.8)	Strength endurance exercise, 6 weeks, 3 × week, 60 min, 3 sets, 15 repetitions, 20% 1RM, compound free weight and resistance machine exercises for lower limb muscles.	↓ CoP ML V (−0.77 cm/s) ↓ CoP AP Area (−0.65 cm^2^) No change in CoP ML Area (−0.1 cm^2^)

SEBT—star-excursion balance test; A—anterior; PM—posterior-medial; PL—posterior-latera1; RM—one-repetition maximum; RPE—rate of perceived exertion. ↑—increase of the respective outcome measure; ↓—decrease of the respective outcome measure.
